# Acknowledgement to Reviewers of *Foods* in 2013

**DOI:** 10.3390/foods3010160

**Published:** 2014-02-27

**Authors:** Foods Editorial Office

**Affiliations:** MDPI AG，Klybeckstrasse 64，CH-4057 Basel，Switzerland

The editors of *Foods* would like to express their sincere gratitude to the following reviewers for assessing manuscripts in 2013:
Achir, NawelBorges, GracileDrago, SilvinaAdriaensen, HansBoysen, LouiseDugan, MikeAl. Marghitas, LiviuBragagnolo, NeuraEller, FredAlmeida, Domingos P.F.Brick, MarkErsoy, BeyzaAmagase, HarunobuCabrita, Maria JoaoEstevinho, LeticiaAnderson, K.L.Cadwallader, KeithEvaristo, IsabelAnsorena, DianaCalvo, LourdesFakhr, MohamedAracil, JoséCandioti, MarioFarid, ChematArai, HirofumiCarcea, MarinaFox, Bradley K.Arlorio, MarcoCasal, SusanaFranz, EelcoAzarpazhooh, ElhamCavallucci, ClaritaGarcia, Coralia V.Azuma, Jun-ichiCavazza, AntonellaGarner, BiancaBaiano, AntoniettaChohan, MagaliGarrido, DoloresBarbaresko, JanettChoi, Young HaeGemtos, TheofanisBarth, Ortrud M.Claps, SalvatoreGenello, LauraBattino, MaurizioCuriel, J.A.Gibis, MonikaBayazit, Senada Cruz, Adriano GomesGil-Muñoz, RocíoBay-Jensen, Anne-ChristineDarko, GodfredGonçalves, Édira C.B.A.Beermann, ChristopherDaymond, AndrewGoorahoo, DaveBekatorou, Argyrode Oliveira Petkowicz, CarmenGottschalk, Ing. KlausBekhit, AladinLúciaGroves, KathyBelobrajdic, Damien P.de Souza, Vanessa RiosGuerra, RuiBernardi, Mag. Ceciliade Wijk, Rene A.Guichard, ElisabethBiswas, DebabrataDinrifo, R.R.Henriques, Marta H.F.Boff, SamuelDomini, Claudia E.Huang, JinBöhm, VolkerDraaijer, ArieHuang, LihuaHwang, Hong-SikMiyashita, KazuoSabo, MirjanaInatsu, YasuhiroMollea, ChiaraSalvador, GarzaJackson, PhilMoodley, RoshilaSato, KenjiJemai, Abdelbasset BessadokMorand, MarionSavary, GéraldineJimenez-Flores, RafaelNicolau, AncaSchmidt Gonçalves, Any Elisa Karrila, Taewee T.Niederman, RobertSouzaKawakita, HidetakaOlmedilla-Alonso, BegoñaSilbergeld, EllenKourkoutas, YiannisOrtega-Rivas, EnriqueSmeds, Annika I.Kozytaska, SvitlanaPanfili, GianfrancoSmith, ScottKulozik, UlrichPanouillé, MaudSouza, Miriam CoelhoLando, AmyPark, Seung-kookStrube, JochenLarsen, Marianne HalbergParsons, Barry J.Su, XiaoLazos, Evangelos S.Pastor, ElenaSun, Yi-MeiLee, ByongPau-Roblot, CorinneSunder Hiranandani, VanmalaLee, Dong SunPerez, Jose LuisSuneetha, VuppuLi, WeiliPerrechil, FabianaSzöllősi, DánielLimbo, S.Piana, LuciaTakasugi, MikakoLin, Sam M.Prieto, MiguelTeixeira, PaulaLorenz, MarioProestos, CharalamposTen Eyck, TobyLu, F.S. HennaQian, MichaelThompson, HenryLutter, PetraRamadan, MohamedTopping, David L.Lutz, MarianeRamanathan, RanjithTortoe, CharlesMackey, BernardRao, U.J.S. PrasadaUslu, HasanMaiani, GiuseppeRathod, PrakashkumarVeneziano, VincenzoMarchello, JohnRatnayake, WajiraVigre, HakanMarsellés Fontanet, RobertRaychaudhuri, Siba P.Wood, DelilahMartini, MinaReaney, Martin J.T.Yoshida, MunehikoMataragas, MariosRerksuppaphol, SanguansakZlatanov, MagdalenMavroudis, Nikolaos E.Rivas, Cristina SolerZorrilla, Susana E.Mehta, Rajendra G.Ross, Mark
Mitra, SuparnaRoutray, Winny



